# Phylogenomics of *Mycobacterium africanum* reveals a new lineage and a complex evolutionary history

**DOI:** 10.1099/mgen.0.000477

**Published:** 2021-02-08

**Authors:** Mireia Coscolla, Sebastien Gagneux, Fabrizio Menardo, Chloé Loiseau, Paula Ruiz-Rodriguez, Sonia Borrell, Isaac Darko Otchere, Adwoa Asante-Poku, Prince Asare, Leonor Sánchez-Busó, Florian Gehre, C. N’Dira Sanoussi, Martin Antonio, Dissou Affolabi, Janet Fyfe, Patrick Beckert, Stefan Niemann, Abraham S. Alabi, Martin P. Grobusch, Robin Kobbe, Julian Parkhill, Christian Beisel, Lukas Fenner, Erik C. Böttger, Conor J. Meehan, Simon R. Harris, Bouke C. de Jong, Dorothy Yeboah-Manu, Daniela Brites

**Affiliations:** ^1^​ I^2^SysBio, University of Valencia-FISABIO Joint Unit, Valencia, Spain; ^2^​ Swiss Tropical and Public Health Institute, Basel, Switzerland; ^3^​ University of Basel, Basel, Switzerland; ^4^​ Noguchi Memorial Institute for Medical Research, University of Ghana, Legon, Accra, Ghana; ^5^​ Centre for Genomic Pathogen Surveillance, Big Data Institute, Nuffield Department of Medicine, University of Oxford, Oxford, UK; ^6^​ Wellcome Sanger Institute, Wellcome Genome Campus, Cambridge, UK; ^7^​ Infectious Disease Epidemiology Department, Bernhard-Nocht-Institute for Tropical Medicine, Hamburg, Germany; ^8^​ Health Department, East African Community (EAC), Arusha, Tanzania; ^9^​ Laboratoire de Référence des Mycobactéries, Ministry of Health, Cotonou, Bénin; ^10^​ Mycobacteriology Unit, Institute of Tropical Medicine, Antwerp, Belgium; ^11^​ London School of Hygiene and Tropical Medicine, London, UK; ^12^​ Mycobacterium Reference Laboratory, Victoria Infectious Diseases Reference Laboratory, Peter Doherty Institute, Melbourne, Victoria, Australia; ^13^​ Molecular and Experimental Mycobacteriology, Research Center Borstel, Borstel, Germany; ^14^​ Partner Site Hamburg-Lübeck-Borstel-Riems, German Center for Infection Research, Borstel, Germany; ^15^​ Centre de Recherches Médicales en Lambaréné (Cermel), Lambaréné, Gabon; ^16^​ Institut für Tropenmedizin, Deutsches Zentrum fuer Infektionsforschung, University of Tübingen, Tübingen, Germany; ^17^​ Center of Tropical Medicine and Travel Medicine, Department of Infectious Diseases, Amsterdam University Medical Centers, Amsterdam Infection and Immunity, Amsterdam Public Health, University of Amsterdam, Amsterdam, The Netherlands; ^18^​ First Department of Medicine, Division of Infectious Diseases, University Medical Center Hamburg-Eppendorf, Germany; ^19^​ Department of Veterinary Medicine, University of Cambridge, Madingley Road, Cambridge, UK; ^20^​ Department of Biosystems Science and Engineering, ETH Zürich, Basel, Switzerland; ^21^​ Institute of Social and Preventive Medicine, University of Bern, Bern, Switzerland; ^22^​ Institute of Medical Microbiology, University of Zürich, Zürich, Switzerland; ^23^​ School of Chemistry and Biosciences, University of Bradford, Bradford, UK; ^24^​ Microbiotica Limited, Bioinnovation Centre, Wellcome Genome Campus, Cambridge, CB10 1DR, UK

**Keywords:** diversity, evolution, genome, mycobacteria, *Mycobacterium africanum*, *Mycobacterium tuberculosis*

## Abstract

Human tuberculosis (TB) is caused by members of the *Mycobacterium tuberculosis* complex (MTBC). The MTBC comprises several human-adapted lineages known as *M. tuberculosis sensu stricto*, as well as two lineages (L5 and L6) traditionally referred to as *Mycobacterium africanum *. Strains of L5 and L6 are largely limited to West Africa for reasons unknown, and little is known of their genomic diversity, phylogeography and evolution. Here, we analysed the genomes of 350 L5 and 320 L6 strains, isolated from patients from 21 African countries, plus 5 related genomes that had not been classified into any of the known MTBC lineages. Our population genomic and phylogeographical analyses showed that the unclassified genomes belonged to a new group that we propose to name MTBC lineage 9 (L9). While the most likely ancestral distribution of L9 was predicted to be East Africa, the most likely ancestral distribution for both L5 and L6 was the Eastern part of West Africa. Moreover, we found important differences between L5 and L6 strains with respect to their phylogeographical substructure and genetic diversity. Finally, we could not confirm the previous association of drug-resistance markers with lineage and sublineages. Instead, our results indicate that the association of drug resistance with lineage is most likely driven by sample bias or geography. In conclusion, our study sheds new light onto the genomic diversity and evolutionary history of *M. africanum *, and highlights the need to consider the particularities of each MTBC lineage for understanding the ecology and epidemiology of TB in Africa and globally.

## Data Summary

Supporting external data includes sequences from studies PRJEB3334, PRJNA52007, PRJEB3223, PRJEB23179, PRJEB5162, PRJEB9680, PRJEB2138, PRJEB7727, PRJNA211633, PRJNA211637, PRJNA211657, PRJNA211658, PRJNA211668, PRJNA211672, PRJNA211700, PRJNA211630, PRJNA211631, PRJNA211648, PRJNA211650, PRJNA211660, PRJNA211665, PRJNA211676, PRJNA211682, PRJNA211702, PRJNA211661, PRJNA211663, PRJNA211711, PRJNA211707, PRJEB27244, PRJEB9545, PRJNA282721, PRJEB27802, PRJNA616081, PRJEB25506 and PRJNA480117, published in DOI:10.1016/S2213-2600(14)70027-X, DOI:10.1038/ng.2744, DOI:10.1038/ng.2878, DOI:10.1038/s41598-018-29620-2, DOI:10.1038/s41598-018-33731-1, DOI:10.1093/gbe/evy145, DOI:10.1371/journal.pone.0052841, DOI:10.1371/journal.pone.0201146, DOI:10.1371/journal.pone.0214088, DOI:10.3201/eid2303.160679 and DOI:10.3389/fmed.2020.00161.

**Impact Statement**

The understanding of *Mycobacterium tuberculosis* genomic diversity and its evolution in Africa, particularly lineage 5 and 6 known as *Mycobacterium africanum *, lags behind our knowledge of other lineages from Europe, North America and Asia. This study fills a research gap in *M. tuberculosis* diversity in Africa, focusing on *M. africanum* lineages, population structure and phylogeography. We have revealed a new lineage (Lineage 9) within *M. africanum* that, unlike the other *M. africanum* lineages, is distributed in East Africa. This finding, together with the recently new lineage found in Central Africa (Lineage 8), starts revealing the hidden diversity of *M. tuberculosis* in Africa. Additionally, our results have provided useful tools for further study of *M. africanum *, including a better understanding of the population structure and robust genetic markers to differentiate lineages and sublineages. Finally, this study has facilitated the inclusion of a strain of the newly described Lineage 9 in a public collection to further facilitate its biological characterization.

## Introduction

Tuberculosis (TB) causes more human deaths than any other infectious disease, and it is among the top ten causes of death worldwide [1]. Among the 30 high TB burden countries, half are in sub-Saharan Africa [1]. TB in humans and animals is caused by the *Mycobacterium tuberculosis* complex (MTBC) [2], which includes different lineages, some referred to as *M. tuberculosis sensu stricto* (lineage 1 to lineage 4 and lineage 7), others as *Mycobacterium africanum* (lineage 5 and lineage 6), a recently discovered lineage 8 [3], as well as different animal-associated ecotypes such as *Mycobacterium bovis *, *Mycobacterium pinnipedii* or *Mycobacterium microti* among others [4, 5]. Some lineages are geographically widespread, while others are more restricted [6]. The latter is particularly the case for lineage 7 (L7), which is limited to the Horn of Africa [7, 8], and L5 and L6, which are mainly found in West Africa [9]. L5 and L6 show a prevalence of up to 50 % among smear-positive TB cases in some West African countries [10–13]. Hence, L5 and L6 contribute significantly to the overall burden of TB across sub-Saharan Africa. Compared to the other MTBC lineages, relatively little is known with regard to the ecology and evolution of L5 and L6 [5, 14]. Two studies have found L5 to be associated with Ewe ethnicity in Ghana [15, 16], supporting the notion that this lineage might be locally adapted to this particular human population [17]. Several epidemiological associations suggest that L6 might be attenuated for developing disease as compared to other lineages (see De Jong *et al*. [9] for a review). For example, L6 has been associated with slower progression from infection to disease [14] and with human immunodeficiency virus co-infection [14, 16], although conflicting data exist [18, 19].

L5 and L6 differ substantially from other MTBC lineages with respect to *in vitro* growth and metabolism [20–25], and in various molecular features relevant for patient diagnosis, such as a non-synonymous mutation in the MPT64 antigen [26] and reduced T cell response to ESAT6 [27]. Mycobacterial genetic determinants are also implicated in virulence and immunogenicity in *M. africanum* [22, 23]. To shed more light on the population genetics, phylogeography and evolutionary history of *M. africanum *, we analysed the largest set of whole-genome data for L5 and L6 generated to date.

## Methods

### *M. africanum* dataset

We analysed 675 genomes to determine the genetic diversity, phylogeography and population structure of *M. africanum* (Table S1, available with the online version of this article). Geographical origin was determined as the country of origin of the patient and when not available the country of isolation. Because the number of different countries was too high to be shown clearly in the figures, and some of them only included very few genomes, we grouped countries together into five African regions following the definitions of Gehre *et al*. [28]: three big regions South, East and Central Africa, and two regions within West Africa, where most of the isolates come from. The western part of West Africa includes Gambia, Senegal, Mauritania, Sierra Leone, Liberia, Guinea, Ivory Coast and Mali, while the Eastern part of West Africa includes Ghana, Nigeria, Benin Niger, Burkina Faso. African maps were built using Mapchart (https://mapchart.net/africa.html)

### Bacterial culture, DNA extraction and whole-genome sequencing

Archived MTBC isolates were revived by sub-culturing on Löwenstein–Jensen medium slants supplemented with 0.4 % sodium pyruvate or with 0.3 % glycerol to enhance the growth of the different lineages and incubated at 37 °C. Five loops full of colonies were harvested at the late exponential phase into 2 ml cryo-vials containing 1 ml sterile nuclease-free water, inactivated at 98 °C for 60 min for DNA extraction using the previously described hybrid DNA extraction method [29]. The MTBC lineages were then confirmed by spoligotyping and long-sequence polymorphisms and sent for whole-genome sequencing.

The MTBC isolates were grown in 7H9-Tween (0.05 %) medium (BD) ±40 mM sodium pyruvate. We extracted genomic DNA after harvesting the bacterial cultures in the late exponential phase of growth using the CTAB (*N*-cetyl-*N*,*N*,*N*-trimethylammonium bromide) method [30].

Sequencing libraries were prepared using a Nextera XT DNA preparation kit (Illumina). Multiplexed libraries were paired-end sequenced on the Illumina HiSeq 2500 (Illumina) system with 151 or 101 cycles when sequenced at the Genomics Facility, ETH Zürich, Basel (Switzerland), HiSeq 2500 (100 bp, paired end) when sequenced at the Wellcome Sanger Institute, or on Illumina MiSeq (250 and 300 bp, paired end) or NextSeq (150 bp, paired end) according to the manufacturer’s instructions (Illumina) when sequenced at the genomics facilities at the Research Center Borstel (Germany).

### Bioinformatics analysis

#### Mapping and variant calling of Illumina reads

The fastq files obtained were processed with Trimmomatic v 0.33 (slidingwindow 5 : 20) [31] to clip Illumina adaptors and trim low-quality reads. Any reads shorter than 20 bp were excluded for the downstream analysis. Overlapping paired-end reads of 15 nucleotides size were merged with SeqPrep v1.2 (https://github.com/jstjohn/SeqPrep). We used bwa v 0.7.13 (mem algorithm) [32] to align the resultant reads to the reconstructed ancestral sequence of *M. tuberculosis* obtained by Comas *et al*. [33]. Duplicated reads were marked by the Mark Duplicates module of Picard v 2.9.1 (https://github.com/broadinstitute/picard) and excluded. To avoid false-positive calls, Pysam v 0.9.0 (https://github.com/pysam-developers/pysam) was used to exclude reads with an alignment score lower than (0.93×read_length)−(read_length×4×0.07), corresponding to more than seven mismatches per 100 bp. SNPs were called with SAMtools v 1.2 mpileup [34] and VarScan v 2.4.1 [35] using the following thresholds: minimum mapping quality of 20, minimum base quality at a position of 20, minimum read depth at a position of 7× and without strand bias. Only SNPs considered to have reached fixation within an isolate were considered (at a within-host frequency of ≥90 %). Conversely, when the within-isolate SNP frequency was ≤10 % the ancestor state was called. Mixed infections or contaminations were discarded by excluding genomes with more than 1000 variable positions with within-host frequencies between 90 and 10 % and genomes for which the number of within-host SNPs was higher than the number of fixed SNPs. Additionally, we excluded genomes with mean read depth <15× (after all the referred filtering steps). All SNPs were annotated using snpEff v4.11 [36], in accordance with the *M. tuberculosis* H37Rv reference annotation (NC_000962.3). SNPs falling in regions such as PPE and PE-PGRS, phages, insertion sequences and in regions with at least 50 bp identities to other regions in the genome were excluded from the analysis, as in the paper by Stucki *et al*. [37]. Customized scripts were used to calculate mean coverages per gene corrected by the size of the gene. Gene deletions were determined as regions with no coverage to the reference genome.

#### Phylogenetic reconstruction and ancestry estimation

All 675 genomes were used to produce an alignment containing only polymorphic sites. The alignment was used to infer a maximum-likelihood phylogenetic tree using the mpi parallel version of RAxML [38]. We used the general time reversible model of nucleotide substitution under the gamma model of rate heterogeneity and performed 1000 alternative runs on distinct starting trees combined with rapid bootstrap inference. To correct the likelihood for ascertainment bias introduced by only using polymorphic sites, we used Lewis correction [39]. The software Treemmer [40] was used to remove redundancy in the collection of 675 whole-genome SNP alignments with the stop option *-RTL* 0.95, i.e. keeping 95 % of the original tree length. The resulting reduced dataset of 424 genomes was kept for subsequent analysis. First, we used the reduced dataset plus a collection of 35 representative animal genomes to produce an alignment containing only polymorphic sites and inferred a maximum-likelihood phylogenetic tree as described above. The best-scoring maximum-likelihood topology is shown. The phylogeny was rooted using '*Mycobacterium canettii*'. The topology was annotated and coloured using the package *ggtree* [41] from R [42] and InkScape.

We inferred the biogeographical histories of L5 and L6 using statistical-dispersal analysis (s-diva) and the Bayesian binary Markov chain Monte Carlo (BBM) method for ancestral state, dispersal-extinction-cladogenesis (DEC), and Bayesian inference for discrete areas (BayArea) implemented in rasp v4.0 [43]. Because we did not have the geographical origin of 18 samples, we used a phylogeny containing only samples from Africa where the isolation or place of birth of the patient was known. The possible ancestral ranges at each node on a selected tree were obtained. For s-diva, the number of maximum areas was kept as two. For BBM analysis, chains were run simultaneously for 500 000 generations. The state was sampled every 100 generations. Estimated Felsenstein 1981 + gamma was used with null root distribution.

#### Population structure and genetic diversity

Genetic structure indices and corrected pairwise SNP differences between the five African regions where genomes are grouped (Western West Africa, Eastern West Africa, Central Africa, South Africa and East Africa) were calculated using Analysis of MOlecular VAriance (AMOVA) using information on the allelic content of haplotypes, as well as their frequencies implemented in Arlequin v3.5.2.2 [44]. The significance of the covariance components was tested using 20 000 permutations by non-parametric permutation procedures.

Pairwise SNP differences and mean nucleotide diversity per site (π) were calculated using the R package *ape* [45]. π was calculated as the mean number of pairwise mismatches among L5 and L6 divided by the total length of queried genome base pairs, which comprise the total length of the genome after excluding repetitive regions (see above) [46]. Confidence intervals (CIs) for π were obtained by bootstrapping (1000 replicates) by re-sampling with replacement the nucleotide sites of the original alignments of polymorphic positions using the function *sample* in R [42]. Lower and upper levels of confidence were obtained by calculating the 2.5th and the 97.5th quantiles of the π distribution obtained by bootstrapping. Population structure was evaluated using principal component analysis (PCA) on SNP differences using R Package *adegenet* [47] and plotted using the *plot* function in R.

To further explore geographical structure, we evaluated the relation between the genomic phylogeny and the geographical origin of the genomes for each lineage separately using linear axis analysis in GenGIS v2.2.2 [48]. The default GenGIS Africa map was used and a maximum-likelihood phylogenetic tree was reconstructed from whole genome SNPs as described above for each lineage separately. A linear axis plot (10 000 permutations) was run at significance level *P* value=0.001. Simpson’s diversity index (D) for geographical diversity was calculated using data from three datasets: (i) the current dataset (*N*=424), (ii) 489 L5 and L6 strains obtained from the sitvit2 database [49], a publicly available database that contains available genotyping (spoligotyping and Mycobacterial Interspersed Repetitive Units - Variable Number of Tandem Repeats), demographic and epidemiologic information on 111 635 clinical isolates, and (iii) 837 genomes genotyped as L5 and L6 from 3580 strains from West Africa [28].

#### Antimycobacterial-resistance-determining mutations and genes

We have used a list of resistance mutations for 11 antibiotics compiled from two independent curated datasets [50] to determine genotypic antimycobacterial resistance. To determinate drug-resistance differences between lineages L5 and L6 and geographical regions, univariate analysis using two-tailed Fisher’s exact test and multivariate logistic regression were performed with R-core packages. We compared any resistance (that is, having at least one resistance markers), and also resistance to three specific drugs independently (rifampicin, ethambutol and isoniazid independently, without considering other resistance markers). South Africa was not considered because it included only one genome.

## Results

### New MTBC lineage: lineage 9

We analysed a total of 675 *M *. *africanum* genomes. These included 350 L5 and 320 L6 genomes, as well as 5 related genomes that could not be classified into any of the known human- or animal-associated MTBC lineages [4, 51]. Out of these 675 genomes, 641 (95 %) came from patient isolates originating in 1 of 21 countries of sub-Saharan Africa. Another 34 (5 %) strains were isolated outside of Africa from patients with an origin other than Africa or unknown (Table S1). To have a representative dataset and avoid overrepresentation of clustered strains, we removed 251 isolates that were redundant, while keeping 95 % of the phylogenetic diversity (>95 % of the tree length) [40]. The resulting non-redundant dataset comprised 424 genomes and showed a similar country distribution compared to the original dataset (Fig. S1).

**Fig. 1.**
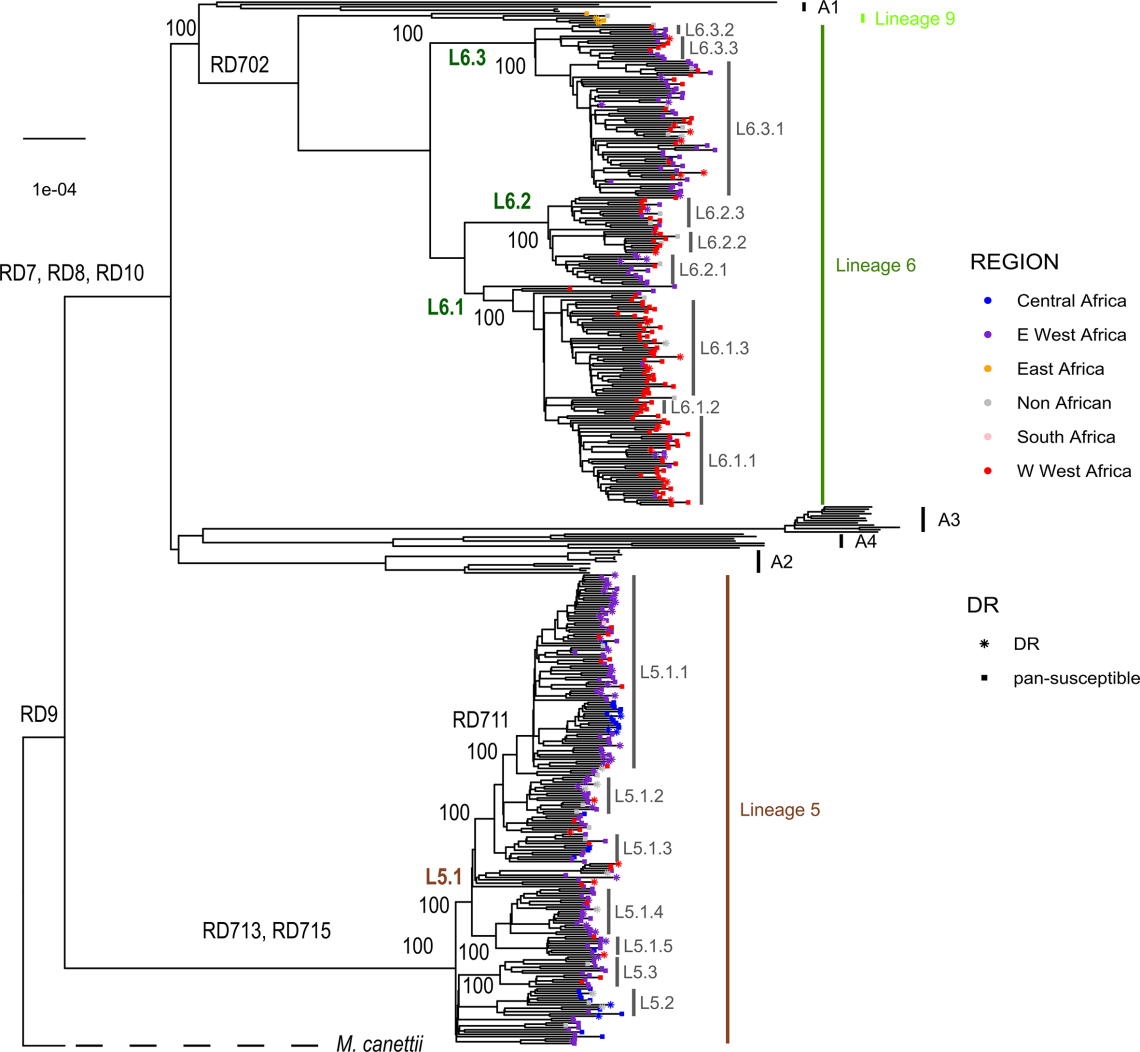
Maximum-likelihood phylogeny of 424 *M *. *africanum* genomes analysed together with animal-associated genomes used as references. Support bootstrap values are indicated at the nodes. The scale bar indicates the number of nucleotide substitutions per site. Nodes are coloured according to country or origin, and the shape of the node indicates susceptible or drug resistance based on absence or presence at least one of the drug-resistance mutations indicated in Table S8.

**Fig. 2.**
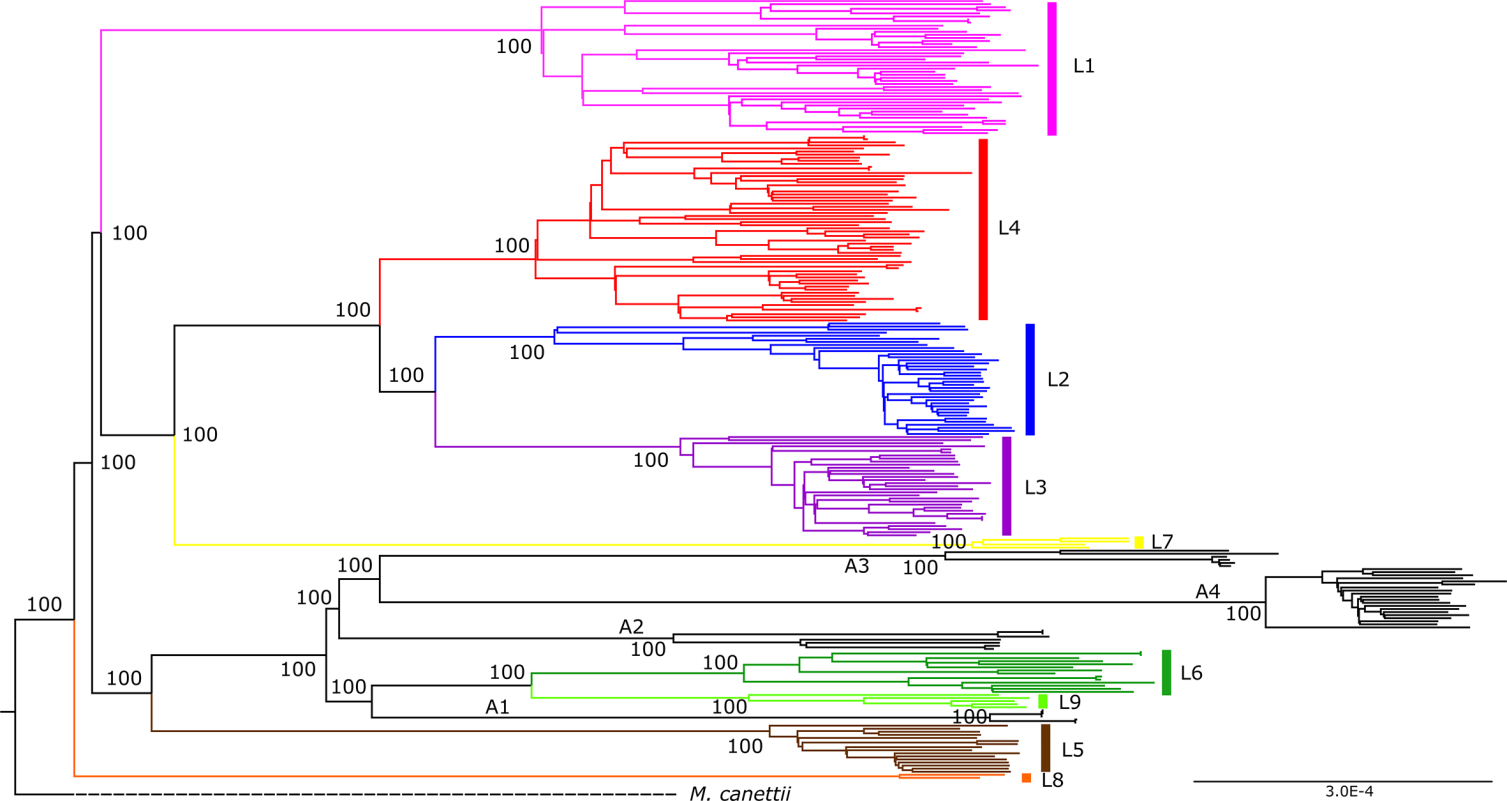
Maximum-likelihood phylogeny of 5 unclassified genomes analysed together with a dataset of 249 MTBC genomes used as references. The five unclassified genomes are coloured in light green and tagged as L9. Animal-associated clades A1 to A4 are indicated and coloured in black. Support bootstrap values are indicated at the deepest nodes. The scale bar indicates the number of nucleotide substitutions per site.

We first focused our analysis on the five genomes that could not be classified into any of the known MTBC lineages. To explore the evolutionary relationship of these five genomes in the context of *M. africanum* diversity, we reconstructed the phylogeny of the 424 *M *. *africanum* genomes together with a dataset of animal-associated MTBC genomes published previously [4]. The resulting phylogeny (Fig. 1) corroborated the separation of L5 and L6, and the localization of L6 in a monophyletic clade together with the animal-associated lineages [4]. To further explore the phylogenetic position of these five genomes, we reconstructed a phylogeny with 248 reference genomes [52], including all eight human-associated lineages and four animal-associated clades (Fig. 2). The five unclassified genomes appeared as a sister clade of L6, branching between L6 and the animal clade A1 (Fig. 2). This L6 sister clade shared deletions with Lineage 6 such as region of difference (RD)702, but did not share other deletions present in animal-associated lineages such as RD1 and RD5.

The geographical origin of the five genomes differed from all other *M. africanum* genomes included in our analysis, as they were the only ones with an origin in East Africa (one from Djibouti, three from Somalia and one isolated in Europe but the patient origin was unknown). By contrast, all L5 and L6 genomes came from isolates from West Africa (354 genomes) or Central Africa (37 genomes), except for 1 isolated from South Africa (Fig. 1) and 28 isolates from outside Africa and of unknown origin.

The five unclassified genomes showed the following *in silico* inferred spoligotype: 772000007775671 (nnnnnnonoooooooooooooooonnnnnnnnnnonnnonnnn) in the genome from Djibouti, 772700000003671 (nnnnnnononnnoooooooooooooooooooooonnnnonnnn) in all three Somalian genomes and a very similar pattern 772600000003631 (nnnnnnononnooooooooooooooooooooooonnnnoonnn) in the genome from Europe. We searched for these three spoligotypes in the international genotyping database SITVIT2, which includes 9658 different spoligotypes from 103 856 strains isolated in 131 countries [49]. Spoligotype 772600000003631 was not found among the 103 856 strains included in the database, and the other two spoligotypes can be considered extremely rare because they have been found only in three strains in the database: 772000007775671 in a strain isolated in France, and 772700000003671 in two strains isolated in The Netherlands, although the patient’s origin is unknown.

The five unclassified genomes showed a mean distance of 1191 SNPs to L6 genomes, 1632 SNPs to L5 genomes and 1491 SNPs to the animal-associated MTBC genomes. Those distances were higher than the corresponding intra-lineage differences: 342 (sd 3.65) within L5, 542 (sd 9.19) within L6 and 332.4 (sd 14.48) within the unclassified genomes. When correcting for the diversity within each lineage, we still found that the five unclassified genomes were separated from the other lineages by 1 294, 582 and 654 SNPs of net distance to L5, L6 and the animal-associated lineages, respectively. The maximum genetic diversity within L9 was 514 SNPs, and occurred between strain G00075 and strain G00074. Conversely, the smallest distance was 99 SNPs between strain G04304 and strain G00075. Given the different geographical distribution and the substantial genetic separation with L6 genomes, we classified these five genomes into a new MTBC lineage that we propose to call MTBC lineage 9 (L9). The strain from L9 corresponding to genome G38445 will be submitted, adding to the original ‘MTBC clinical strain reference set’ [53], to the mycobacteria culture bank of the Belgian Co-ordinated Collections of Micro-organisms (BCCM/ITM).

We looked for deleted regions in the L9 genomes that could be used as phylogenetic markers, as was done for other MTBC lineages in the past [6, 54, 55]. We identified one region deleted in all L9 genomes that spanned from Rv1762c to Rv1765. However, this region is not a robust phylogenetic marker because (i) Rv1763 and Rv1764 are putative transposases, and (ii) partially overlapping deletions can be found in other lineages. Specifically, Rv1762c was deleted in '*Mycobacterium orygis*', and the region between Rv1763c and Rv1765 was deleted in L6 genomes. Hence, instead, we report a list of SNPs that can be used as phylogenetic markers for L9 (Table S2) given that they appear in all five L9 genomes and are absent from genomes from other lineages [40]. Given the low number of L9 genomes, we focused the remainder of our analysis on *M. africanum* L5 and L6.

### Sublineages within L5 and L6

Our extended genomic analysis of L5 and L6 confirmed the deletions of the previously described RDs, including RD7, RD8, RD9 and RD10 [54, 55], and RD713 and RD715 [6], as indicated in the phylogeny (Fig. 1). However, the deletion of RD711 could not be confirmed as a L5 marker as proposed previously [6], as it was only deleted in a subset of L5 genomes, as reported recently [22]. We found RD711-deleted genomes to form a monophyletic clade within L5 (Fig. 1); named L5.1.1 considering previous nomenclature as proposed by Ates *et al*. [22]. In contrast, RD702 was found to be deleted in all L6 strains, as shown previously [6], as well as in the newly defined L9 strains (Fig. 1).

**Fig. 3.**
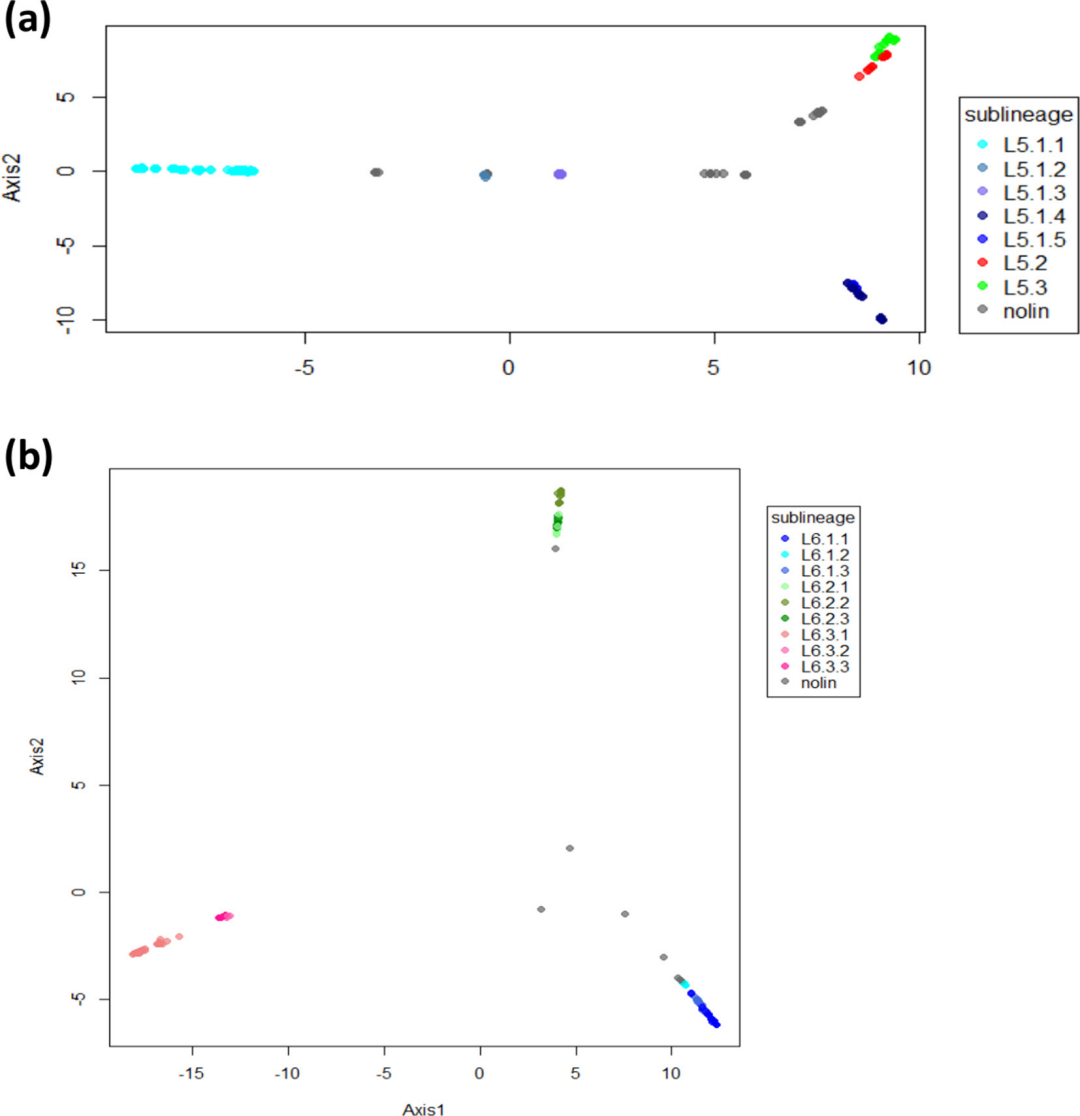
PCA based on genomic variable SNPs. The PCA was conducted separately for L5 (a) and L6 (b). Colours indicate different sublineages and grey indicates genomes with no sublineage assigned ‘nolin’.

Our phylogeny revealed a different topology for L5 compared to L6. Specifically, the L5 phylogeny showed little structure. Nevertheless, we subdivided L5 into three main sublineages that were well differentiated and highly supported by bootstrap values >90, and named them consistent with previous nomenclature [22] as L5.1, L5.2 and L5.3. Due to the high genomic diversity within L5.1, this group was further subdivided into five main sublineages (Fig. 1), leading to a total of seven L5 sublineages. Sublineage classification was only partially corroborated by the results of the PCA performed on whole-genome SNPs (Fig. 3a). By contrast, L6 showed a more differentiated population structure with three clearly differentiated monophyletic main sublineages (L6.1, L6.2 and L6.3) that could be further subdivided into at least three other sublineages each, to a total of nine L6 sublineages (Figs 1 and 3b). The main three L6 sublineages L6.1, L6.2 and L6.3 were also clearly separated using PCA unlike the sub-divisions within each sublineage (Fig. 3b). To explore the robustness of the classification beyond PCA, we estimated genetic differentiation for each of these sublineages using the fixation index (FST) based on Wright’s F-statistic [56] as a measure of population differentiation due to genetic structure. We conducted a hierarchical analysis comparing the population structure at the two levels of subdivision: one level with the three main sublineages for both L5 and L6, and a second level with all seven and nine sublineages of L5 and L6, respectively. The L5 population structure showed the highest differentiation within all seven sublineages, where the highest population differentiation index FST=0.48 (*P* value <0.000001), and the lowest population differentiation index was found between the three main sublineages (FST=0.14, *P* value=0.04915). Similarly, FST between all seven L5 sublineages showed moderate differentiation with pairwise FST values between 0.3 and 0.5 (Table S3), and net pairwise differences between 76 and 206 SNPs (Table S4). Conversely, for L6, the higher differentiation was between the three main sublineages (L6.1, L6.2, L6.3, with 47 % of the variation, FST=0.47, *P*=0.0035), mirroring the PCA results. The differentiation between all nine sublineages of L6 was also stronger than for L5, with FST values ranging between 0.25 and 0.75 (Table S5), and net pairwise differences of between 73 and 493 SNPs (Table S6). A list of SNPs found exclusively in each of the L5 and L6 sublineages is shown in Table S7.

In summary, different metrics point to a stronger population sub-division of L6 than L5. We propose to divide L6 in three main sub-lineages (L6.1, L6.2, L6.3), which in turn can be sub-divided in three sub-groups each (Fig. 1). For L5, we propose three new sub-groups within L5.1 [22] (Fig. 1) and a new main sublineage (L5.3).

### Phylogeography

To explore the phylogeographical structure of L5, L6 and L9, we mapped the geographical origin of the genomes onto the phylogenetic tree as a coloured point at the end of each branch (Fig. 1). We grouped the different countries represented in the dataset into five regions in Africa: East, South and Central, and the Western part of West Africa (^W^West Africa) and the Eastern part of West Africa (^E^West Africa). We observed that most sublineages in L6 showed a characteristic geographical association at the regional level. At least five sublineages within L6 (all three L6.1 and two L6.2) showed a majority of genomes originating in ^w^West Africa, mostly The Gambia. By contrast, genomes from one sublineage within L6.2, from all three L6.3 sublineages and a few scattered L6 genomes from other sublineages came from ^E^West Africa, mostly Ghana. Only a few L6 strains were found in Central Africa (*N*=2) or outside Africa (*N*=15). However, we did not detect such phylogeographical structure for L5 sublineages, with most genomes originating in ^E^West Africa (mostly Ghana), just two sublineages (L5.2 and one sublineage within L5.1.1) in Central Africa and only a few dispersed genomes originated from ^W^West Africa.

**Fig. 4.**
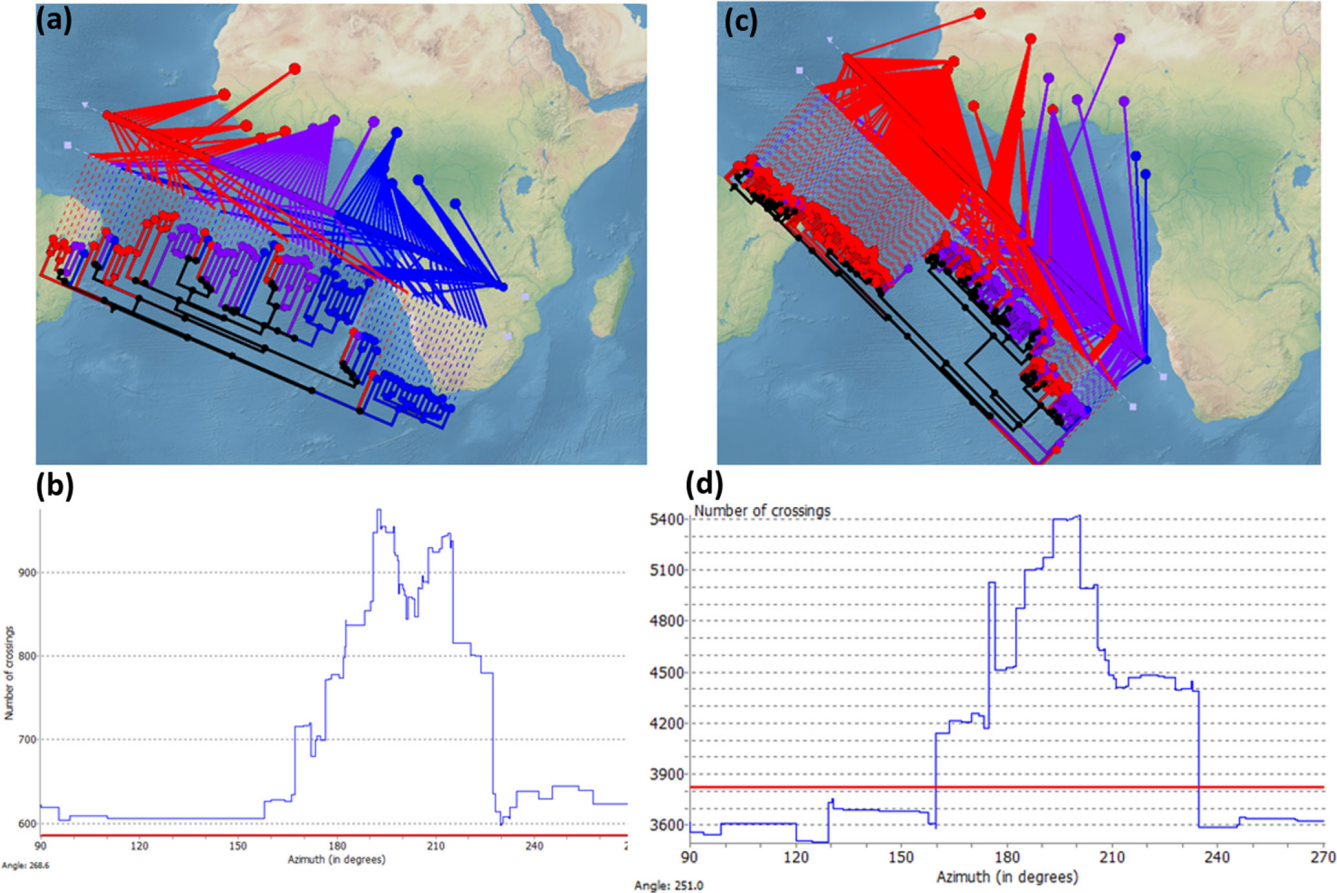
Phylogeographical structure in L5 and L6. Linear axis plot between the genomic phylogeny and the geographical origin of the genomes for L5 (a) and L6 (c), with minimum crossing between each leaf node in the phylogeny and its geographical distribution. Histograms show the number of crossing for each inclination of the axis, and the red lines indicate the number of crossings expected by chance for L5 (b) and L6 (d).

To better understand the different geographical substructure within L5 and L6, we conducted an independent phylogeographical analysis using the GenGIS software, where each whole-genome SNP phylogeny was superimposed onto the five main African regions defined previously (Fig. 4a, c). If there is geographical separation, we expect the geographical distribution of the genomes to fit the phylogenetic tree structure. Fitting the tree is determined by finding a linear axis where the ordering of leaf nodes matches the ordering of sample sites according to the geographical distribution of each leaf node. If we draw a line between each leaf node in the phylogeny and its geographical distribution, a perfect match will result in minimum crossing of lines between the phylogeny and the map. Consequently, marked phylogeographical structure will result in significantly less crossing than the number of crossings expected by chance. We found several orientations of the tree’s geographical axis resulting in less crossings than expected by chance in L6 (*P* <0.001, 10.000 permutations; blue points below the red line in Fig. 4d). By contrast, for L5 we did not find less crossing than expected by chance (no blue points below the red line in Fig. 4b). These results indicate a marked geographical structure within L6, but not within L5.

To further confirm the different phylogeographical structures within L5 and L6, we calculated population differentiation indices considering each African region as a different population for each lineage. This analysis revealed some phylogeographical substructure within L6, where the percentage of variation attributed to different regions within Africa was 15 % (FST=0.15, *P* <0.00001). By contrast, L5 did not show any well-marked population differentiation, as the percentage of the variance attributed to population differences was only 6.6 %, with the rest of the variation attributed to intra-population differences (FST=0.036, *P* <0.00001). This result further supports the observation of higher geographical structure within L6 than L5.

Finally, we explored possible differences in geographical range. Our dataset was geographically biased because it was designed to assemble as many L5 and L6 genomes from as many countries as possible. Therefore, we analysed our genome dataset together with two other large datasets where samples were not genome sequenced but genotyped using spoligotyping, and compared the geographical distributions of L5 and L6 [28, 49]. This combined dataset included *N*=733 L5 from 27 African countries and *N*=1031 L6 from 18 African countries. We expected that a broader geographical distribution of a specific lineage is associated with a lower probability that two individuals selected randomly will belong to the same country. We used the Simpson’s Index (D) to measure the probability that two individuals randomly selected from a sample will belong to the same country. We found a larger diversity of countries of origin in L5 than in L6 (D=0.16 vs D=0.27), indicating a broader geographical distribution of L5.

These results as whole indicate that L5 has generally expanded more within West Africa than L6. In the latter, the population sub-division described in the previous section reflects a stronger association between phylogenetic groups and geographical regions reflecting more restricted expansions.

### Ancestral geographical distribution of L5, L6 and L9

**Fig. 5.**
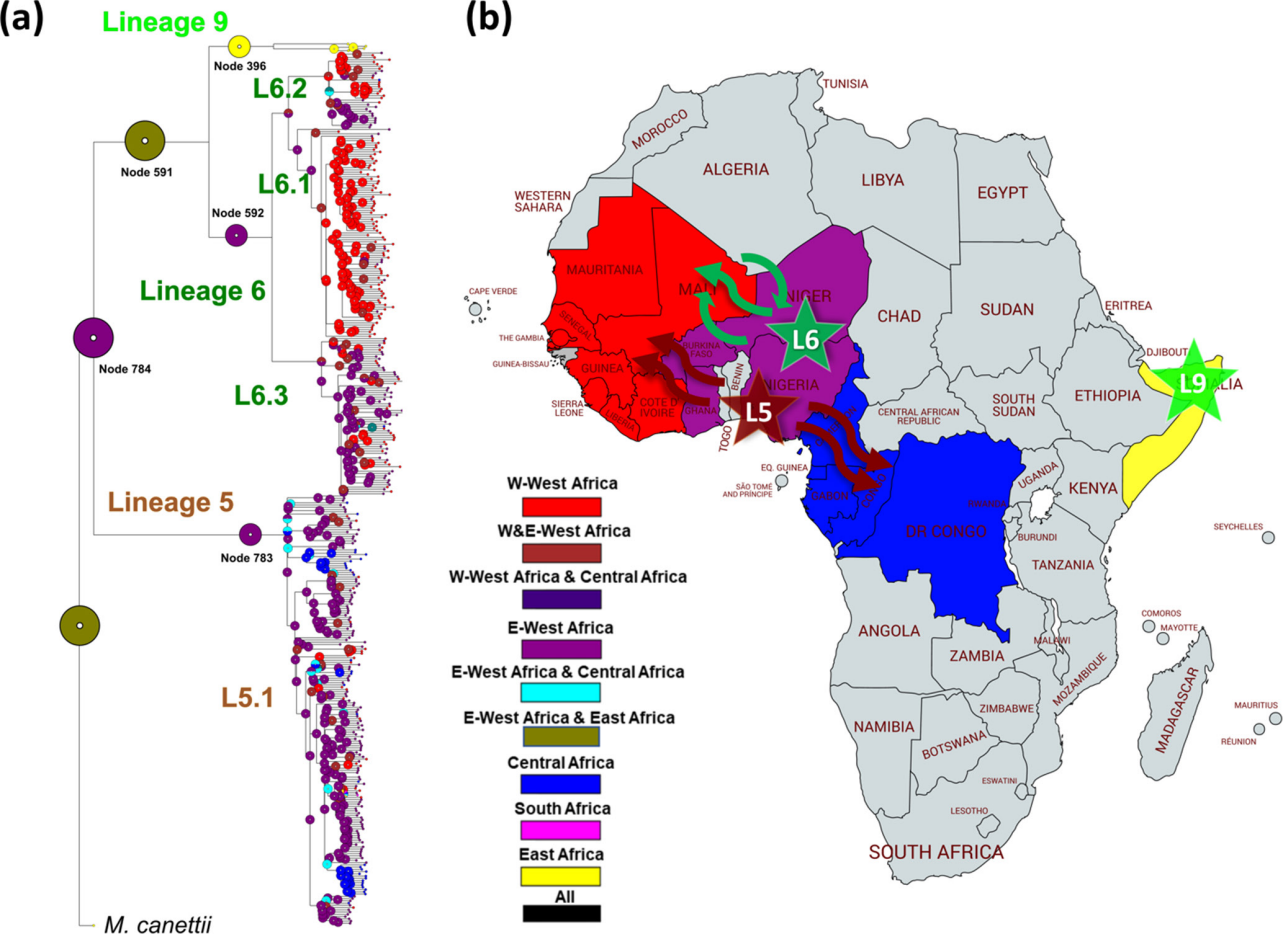
Geographical ancestral distributions of L5, L6 and L9. (a) Ancestral area reconstruction by the Bayesian binary model onto the maximum-likelihood phylogeny. Circles represent the probabilities of ancestral ranges, and the most likely ancestral areas are indicated by their corresponding colour codes. (b) The four geographical areas considered in this analysis are coloured in the map, the most likely ancestral areas for each lineage are shown as stars, and movements of strains inferred from phylogeny indicated as arrows. The map was created using Mapchart (https://mapchart.net/africa.html).

Next, we explored the most likely geographical origin of L5 and L6 using four methods based on a Bayesian approach [43]. The probabilities of ancestral distribution areas for the principal nodes were always congruent with at least two methods, but the results of the two other methods were either inconclusive or showed minor discrepancies (Figs 5a and S2). For L5, two of the four methods inferred ^E^West Africa as the most likely origin (marginal probability was 1.0 using both Bayesian binary and s-diva), while the other two were inconclusive (marginal probabilities were ^E^West–Central, 0.94 and 0.58 with BayArea and DEC, respectively; node 783 in Figs 5a and S2). For L6, two methods also pointed to ^E^West Africa as the most likely origin (0.77 and 1.0, of marginal probability using Bayesian binary and s-diva, respectively) and two methods supported both regions of West Africa as equally likely (0.94 and 0.58 using BayArea and DEC, respectively; node 592 in Figs 5a and S2). The ancestral distribution of L9 was predicted to be East Africa based on all four methods (node 396 in Figs 5a and S2).

The ancestral distribution of the common ancestor between L6 and L9 was not confidently predicted because marginal probabilities supported similarly ^E^West Africa (0.65 and 0.57 using BBM and DEC (node 591 Figs 5a and S2) and both regions within West Africa (0.5 using s-diva and BayArea). By contrast, the ancestral distribution for the common ancestor of L5, L6 and L9 showed more consistency, where ^E^West Africa was supported by three methods (0.74, 1.0 and 0.57 using s-diva, BBM and DEC, respectively) and only one method predicting both ^E^West Africa and East Africa with a marginal probability of 0.99 (BayArea: node 784, Figs 5a and S4).

In summary, ^E^West Africa might have played an important role as the origin *M. africanum* L6 and L5, while L9 has a clear ancestral geographical distribution in East Africa. Although very strong statistical support is missing, our inferences point to a common ancestor of all *M. africanum* L5, L6 and L9 initially originating in West Africa.

### Differences in genetic diversity between lineages

**Fig. 6.**
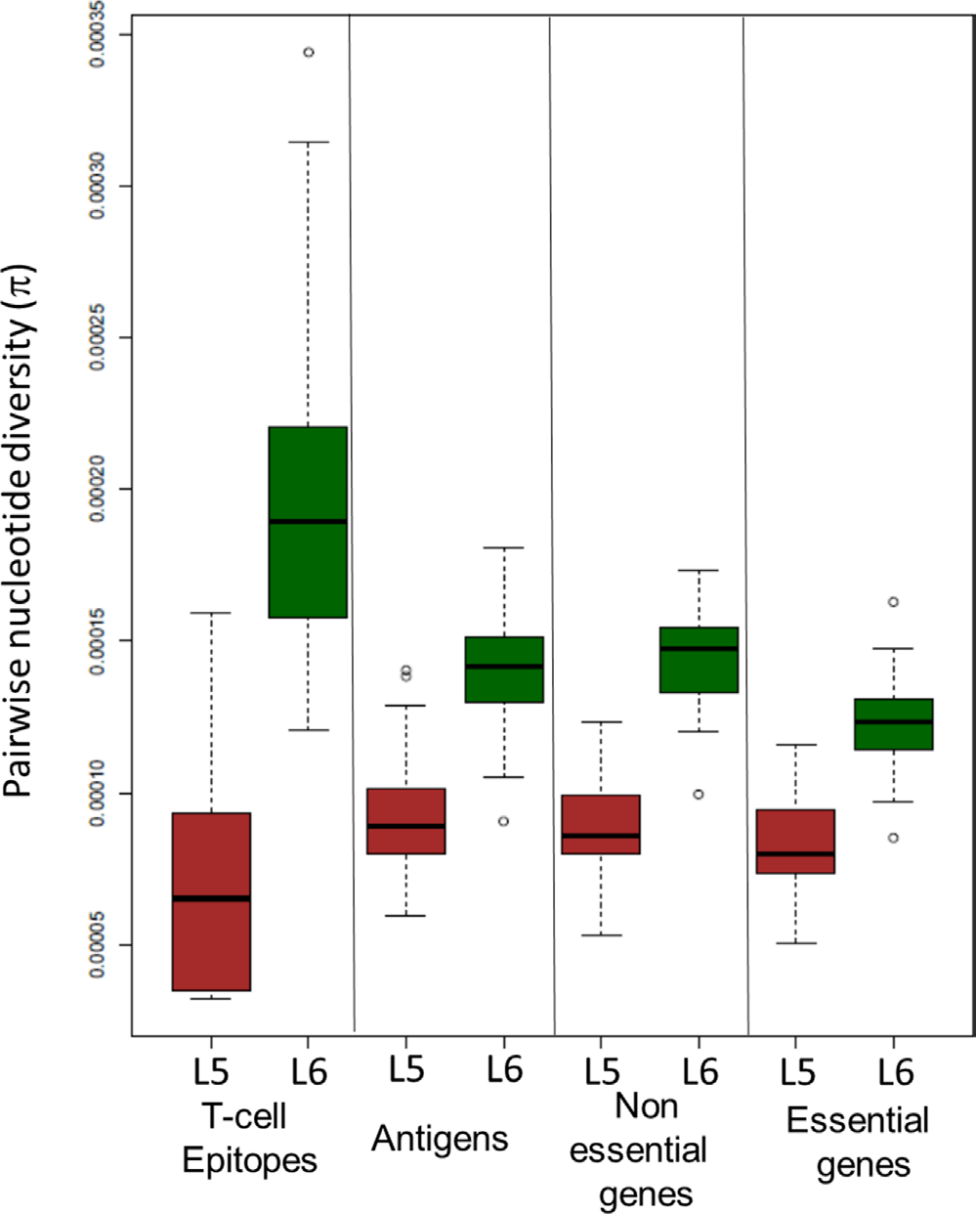
Nucleotide diversity (π). Comparison of pairwise nucleotide diversity (π) between L5 and L6 across gene categories

In support of our previous findings based on a more limited dataset [57], we found that L6 was more genetically diverse than L5 with a significantly higher number of SNPs between pairs of sequences (median values 553 vs 321; *P* value <2.2×10^−15^), and significantly higher mean nucleotide diversity (1.4×10^−4^ vs 8.7×10^−5^; *P* value <2.2×10^−15^). To explore whether this trend was consistent across the whole genome, we assessed the nucleotide diversity in different regions that might be under different selection pressures: essential genes, non-essential genes, antigens and T cell epitopes (Fig. 6). Although the genetic diversity was higher in all these different gene categories for L6 (Fig. 6), epitopes showed an inverted pattern in diversity between lineages (Fig. 6). Specifically, epitopes in L6 showed significantly higher genetic diversity than non-essential genes (Wilcoxon signed rank test *P* value <2.2×10^−15^), while the opposite was found for L5, with epitopes showing significantly lower genetic diversity than non-essential genes (Wilcoxon signed rank test *P* value <2.2×10^−15^).

### Drug-resistance mutations

Antibiotic pressure is a strong selective force in bacteria including the MTBC. Hence, we explored the difference in drug-resistance determinants between L5 and L6. We found that among the 424 genomes analysed, 89 (21 %) showed at least one genetic marker of antimycobacterial-drug resistance, with 24 (6 %) being multi-drug resistant (defined as resistance to at least isoniazid and rifampicin; Table S8). The most common resistance marker found was for streptomycin, with 60 genomes showing 13 different resistance-conferring mutations. The next most common was resistance to rifampicin and isoniazid, with 32 and 29 genomes, respectively. Additional resistance was found to ethambutol, fluoroquinolones, ethionamide, pyrazinamide and aminoglycosides (Table S8). L5 genomes were more likely than L6 genomes to carry mutations associated with any resistance in a univariate analysis [odds ratio (OR) 1.76, 95 % CI 1.08–2.92, *P* value=0.0168], but that association disappeared once the different geographical regions were taken into account in a multivariable analysis (Table 1). In particular, ^E^West Africa genomes were associated with the presence of any resistances (OR 12.35, 95 % CI 6.64–22.97, *P* value <0.001; Table 1), with rifampicin resistance (OR 11.57, 95 % CI 4.54–29.47, *P* value <0.001; Table S9) and isoniazid resistance (OR 7.73, 95 % CI 3.15–17.00, *P* value <0.001; Table S9). Non-African genomes were associated with any resistances (OR 5.47, 95 % CI 2.43–13.52, *P* value <0.001; Table 1) and isoniazid resistance (OR 5.11, 95 % CI 1.75–14.90, *P* value <0.001; Table S9). L5 was negatively associated with resistance to rifampicin in a univariate analysis (OR 0.31, 95 % CI 0.11–0.78, *P* value=0.00924; Table S9), but not isoniazid (OR 0.71, 95 % CI 0.36–1.38, *P* value=0.222; Table S9). However, as before, these associations seem to be driven by geographical regions, as shown in the multivariate analysis (Table S9). Contrary to a previous report by Ates *et al*. [22], we found no evidence of differences in drug-resistance genotype between L5.2 and other L5 genomes (OR 1.21, 95 % CI 0.36–4.11, *P* value=0.49; Fisher’s exact test).

**Table 1.** Association of genotypic resistance (presence of at least one resistance marker to one drug) with lineages and geographical region South Africa was not included because it includes one genome. * indicates reference category.

Lineage/region	No. (%) with DR (total *N*=87)	No. (%) with no DR (total *N*=580)	Univariate regression		Multivariate regression	
			OR (95 % CI)	*P* value	OR (95 % CI)	*P* value
**Lineage**						
L6*	31 (35.6)	287 (49.5)	–	–	–	–
L5	56 (64.4)	293 (50.5)	1.76 (1.08–2.92)	0.0168	0.85 (0.48–1.50)	0.589
**Region**						
^W^West Africa*	34 (39.1)	465 (80.2)	–	–	–	–
Central Africa	7 (8.0)	49 (8.4)	1.95 (0.82–4.64)	0.129	2.11 (0.84–5.26)	0.108
^E^West Africa	37 (42.5)	44 (7.6)	11.50 (6.57–20.11)	<0.01	12.35 (6.64–22.97)	<0.01
Non-African	9 (10.3)	22 (3.8)	5.59 (2.39–13.09)	<0.01	5.74 (2.43–13.52)	<0.01

DR, Drug resistance marker.

## Discussion

*M. africanum* has traditionally been considered a single entity and a separate species from what classically has been referred to as *M. tuberculosis sensu stricto*. The results presented here provide novel insights into the genomic particularities of the different lineages within *M. africanum *: L5, L6 and a new group described in this study, L9. Differences between these three lineages further emphasize the need to consider these lineages as separate phylogenetic and ecological variants within the MTBC.

Unexpectedly, our study of the global diversity of *M. africanum* revealed the presence of another MTBC lineage in Africa: L9, which is genetically close to L6. Unlike L5 and L6, which predominately occur in West Africa, L9 seems to be restricted to the East of Africa. Given that only five L9 isolates were included in our study, future studies are needed to confirm this observation [7, 8]. In this respect, L9 is similar to L7 and the recently described L8 [3], which are also mainly restricted to East Africa, but genetically more distant. L9 strains are also not part of a group of strains classified previously as *M. africanum* type II (East African clade), which belong to L4 and were erroneously thought to be *M. africanum* [58]. We found clinical strains of L9 to be rare compared to L5 and L6, and this observation also resembles the situation for L7 and L8. We cannot dismiss that this might be due to limited sampling, but the observation that clinical strains from L7, L8 and L9 originate in East Africa and are generally rare, while L5 and L6 are more prevalent and distributed across West and Central Africa, raises the question of whether the reduced prevalence of L7, L8 and L9 is due to biological reasons, or social-environmental causes that render L7, L8 and L9 to be less successful. The lack of experimental and epidemiological data on L7, L8 and L9 impedes a profound discussion on the matter. However, the fact that L9 is genetically closer to L6 and L5 than to L7 and L8 speaks against a common intrinsic biological determinant shared by L7, L8 and L9. Instead, convergence in the biology of the strains and/or in the socio-demography of the host is a more likely driver of the evolutionary history of L7, L8 and L9.

Our phylogeographical analyses mostly suggested that the common ancestors of L5 and L6 lived in ^E^West Africa. We inferred that several subgroups of L5 moved from ^E^West Africa to Central Africa, while L6 subgroups moved mostly within West Africa. One of these events resulted in half of the L6 genomes in our dataset representing strains that moved from ^E^West Africa to ^W^West Africa and with few dispersals back to ^E^West Africa (Fig. 5b). The ancestral reconstruction of L6 and L9 did not provide any clear signal, with ^E^West Africa and East Africa equally supported. For the ancestral distribution of all *M. africanum,* there was no consensus, but three out of four methods agreed on ^E^West Africa being the most likely place of origin. That would imply that L5 and L6 diversified there, and L9 migrated to East Africa. Because '*M. canettii*', the most closely related species of *M. tuberculosis* is restricted to East Africa, we and others have proposed that East Africa is the likely origin of the MTBC [59–61]. If confirmed, the current geographical distribution of L5, L6 and L9 could be explained by a migration of their common ancestor from East Africa to West Africa, with the ancestor of L9 then moving back to East Africa. Unfortunately, the region of Central Africa is very poorly represented in our dataset. Possibly having more representatives from this area, which makes the transition between East and West Africa, could bring new and relevant insights into the history of *M. africanum* and L9. Additionally, clade A1, due to its phylogenetic positioning, could potentially bring insights into the phylogeography of *M. africanum.* However, clade A1 as is currently known, contains only animal-adapted MTBC for which very few representatives are known. The geographical range of these non-human pathogens is poorly described, with one member (the ‘chimp bacillus’) isolated in West Africa, and the remaining members isolated in meerkats, mongooses and hyraxes in Southern Africa. As we have discussed in a previous work [4], probably the geographical range of these pathogens is broader than what is currently known, and including them in our geographical analysis would not inform particularly well our inferences and would, at the same time, put weight on Southern Africa.

The work presented here also demonstrates differences in the population structure of L5 compared to L6. While L6 showed a marked phylogenetic structure comprising distinct sublineages associated with different geographical regions, the classification of L5 into sublineages was not so clearly supported, despite the broader geographical range of L5 compared to L6. However, an independent study supported the split of L5.3 into L5.3.1 and L5.3.2 due to considerable gene content variability [62].

Our work confirms previous observations, where L6 shows a higher genomic diversity compared to L5 [57]. In particular, human T cell epitopes in L6 were more diverse than non-essential genes, while the opposite was true for L5. Several studies have shown that human T cell epitopes in the human-adapted MTBC are overall more conserved than non-essential genes [33, 63, 64]. This observation gave rise to the hypothesis that the MTBC might benefit from T cell recognition that drives lung pathology, leading to enhanced bacterial transmission [65]. The fact that L6 differs in this respect from L5 and the other human-adapted MTBC lineages indicates a potential different ecological niche, including possible animal reservoirs [12], which would also be supported by the phylogenetic proximity of L6 to the animal-adapted lineages of the MTBC (Fig. 1). Moreover, human TB caused by *M. bovis* compared to *M. tuberculosis* has also been associated with human immunodeficiency [66] and higher levels of immunosuppression [67], which also suggest that L6 might be an opportunistic pathogen, similar to *M. bovis* in humans [68].

We found L5 genomes more likely to carry any drug resistance-conferring mutations than L6 only in a univariate analysis. However, this observation was driven by genomes from Ghana, where L5 dominates. In univariate and multivariate analysis, genomes from ^E^West Africa, independently of lineage, were associated with genotypic resistance to any drug, rifampicin and also isoniazid resistance. Previous findings from Ghana, found L5 associated to *inhA* promotor mutations conferring resistance to isoniazid compared to L4. However, in our study, we did not find L5 associated to *inhA* promotor region with isoniazid (OR 1.83, 95 % CI 0.46–7.84, Fisher exact test *P* value=0.37). In addition, contrary to the previous study from Ates *et al.* [22] based on a smaller dataset, our larger sampling indicated no association between drug resistance and a specific sublineage of L5 [22]. Our main study limitation is sampling bias, leading to an overrepresentation of isolates from the Gambia and Ghana. Consequently, drug-resistance genotype differences found are more likely to have been driven by a sampling bias of drug-resistance isolates in different countries rather than differences in control programmes.

The overrepresentation of genomes from the Gambia and Ghana could contort our observation regarding genomic diversity and population structure too. Moreover, including more genomes from other countries will likely reveal additional sub-lineages within L5 and L6.

In summary, we describe a large-scale whole-genome sequencing and a comprehensive phylogenomic analysis of clinical isolates classically referred to as *M. africanum* from 21 countries across Africa. Our findings have resolved hidden diversity, a complex evolutionary history and different patterns of variation between lineages. Our results contribute to a better understanding of the MTBC lineages restricted to parts of Africa. These findings might assist in unravelling the molecular signatures of adaptations, and inform the development of targeted interventions for controlling TB in that part of the world.

## Supplementary Data

Supplementary material 1

Supplementary material 2
