# Supplementary material for: Phylogenomics of Mycobacterium africanum reveals a new lineage and a complex evolutionary history
Source: Microb Genom. 2021 Feb 8;7(2):000477. doi: 10.1099/mgen.0.000477 (PMC8208692; doi:10.1099/mgen.0.000477)
Supplement: Supplementary material 2 [file mgen-7-477-s002.pdf]

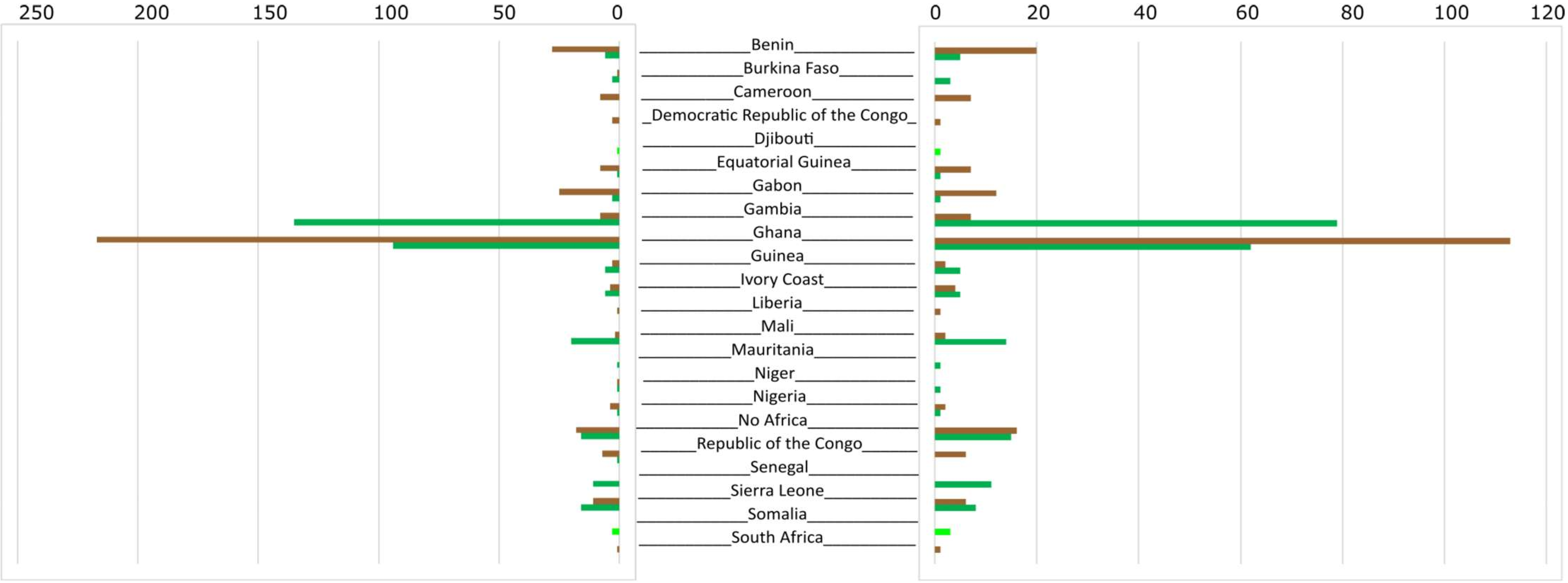


**Fig. S1. Lineage and country distribution.** Genomes analysed for the initial dataset (A) and the non-redundant dataset (B). L5 genomes are indicated in brown bars, L6 genomes in green bars and L9 genomes in light green bars.

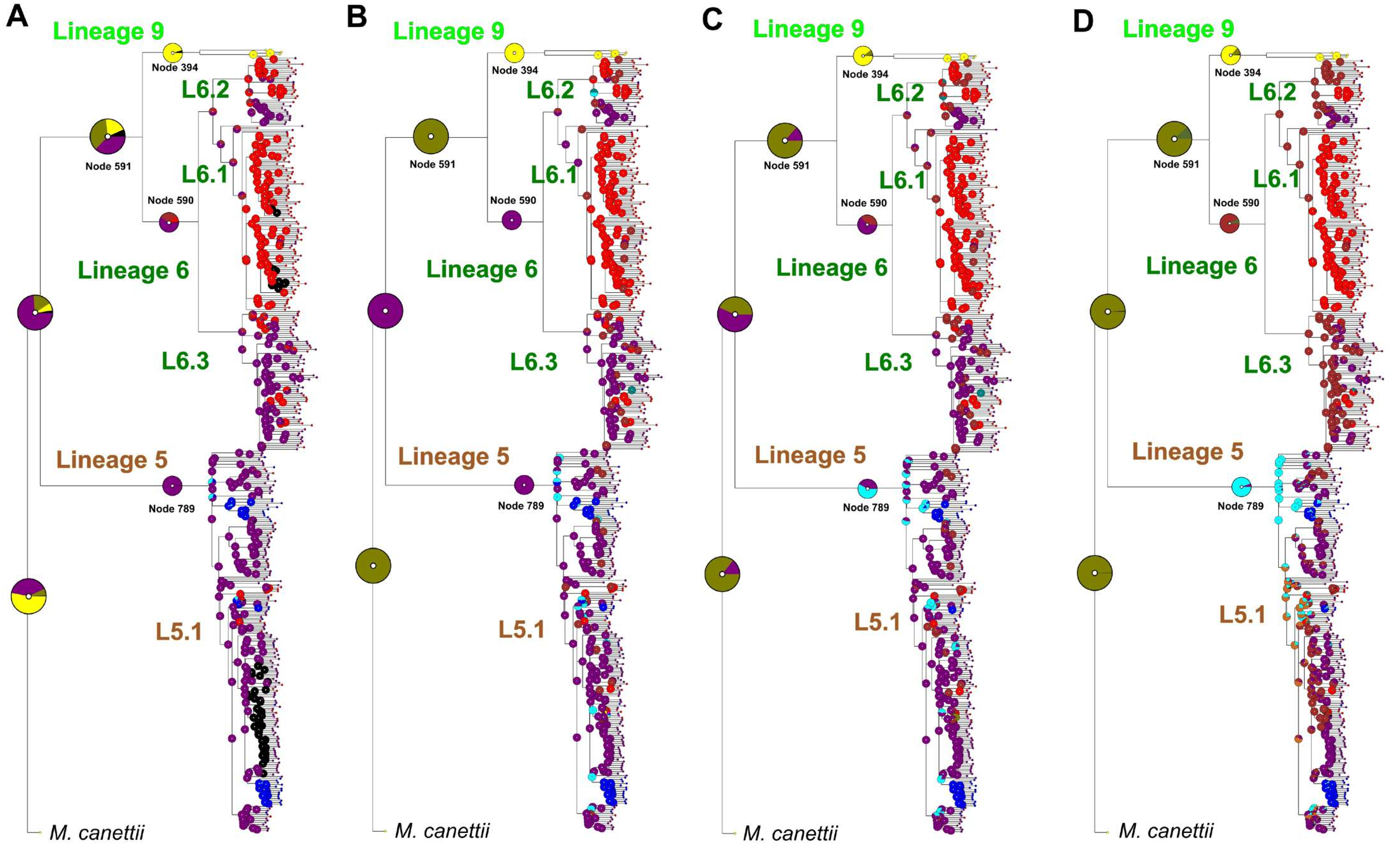


**Fig. S2. Ancestral area reconstruction onto the maximum likelihood phylogeny.** Circles represent the probabilities of ancestral ranges, and the most likely ancestral areas are indicated by their corresponding color code.
The inset map represents the four geographical areas considered in this analysis.
Results for all four methods are shown: Bayesian binary (A), DIVA (B), DEC (C) and BayArea (C).
